# A Neurocognitive Perspective on the Forms and Functions of Autobiographical Memory Retrieval

**DOI:** 10.3389/fnsys.2019.00004

**Published:** 2019-01-29

**Authors:** Signy Sheldon, Can Fenerci, Lauri Gurguryan

**Affiliations:** Department of Psychology, McGill University, Montreal, QC, Canada

**Keywords:** autobiographical memory, retrieval orientation, hippocampus, episodic memory, component processing, memory construction, dual processing systems, default mode activity network

## Abstract

Autobiographical memory retrieval involves constructing mental representations of personal past episodes by associating together an array of details related to the retrieved event. This construction process occurs flexibly so that the event details can be associated together in different ways during retrieval. Here, we propose that differences in how this association occurs support a division in autobiographical remembering. We first review theories of autobiographical memory organization that suggest that episodic details of an experience are processed along a gradient of abstraction. This organization allows for the same autobiographical event to be recalled as either a conceptualized or perceptually-based episodic memory. We then use neuroimaging evidence to show how this division within episodic autobiographical memory is also present in the brain, both at a network level and within the hippocampus. Specifically, we suggest that the anterior and posterior hippocampus are obligatorily tuned towards constructing conceptual vs. perceptual episodic representations of autobiographical memories. Finally, we discuss the directive purpose of this proposed division of episodic remembering by reviewing decision scenarios that benefit from recalling the past as a conceptual vs. a perceptual episode. Conceptual remembering is useful to guide ambiguous decisions that have yet to be encountered whereas perceptual remembering is useful to guide decisions for well-structured tasks that have been previously experienced. We emphasize that the ability to shift between conceptual and perceptual forms of remembering, by virtue of hippocampal specialization, during decision-making and other memory-guided actions is the key to adaptive behavior.

## Introduction

Autobiographical memory is often described in terms of two types of long-term memory, semantic (knowledge about the self) and episodic (event-specific knowledge related to past personal experiences) memory (Tulving, 2002). The episodic memory component is considered the defining feature of autobiographical memory retrieval as it allows for past events to be remembered in rich detail (Conway, 2001; Rubin, 2005). When remembering, episodic memory processes actively reconstruct an autobiographical experience by associating together different experiential details, including the perceptual and conceptual elements (Bartlett, 1932; Schacter and Addis, 2007; Schacter et al., 2011; Sheldon and Levine, 2016). Here, we propose that this reconstructive characteristic of episodic memory allows for different forms of autobiographical remembering by constructing memory representations with different combinations of details. Specifically, we suggest that autobiographical events can be represented and remembered as conceptual or perceptual experiences and that these forms: (a) rely on different neural mechanisms; and (b) contribute to different functions of memory, particularly when memory is used to solve a current problem or direct a future action (e.g., Vandermorris et al., 2013; Madore et al., 2016; Schacter et al., 2017; Mar and Spreng, 2018).

In the sections to follow, we expand on these two points by exploring the nature and reasons for a perceptual/conceptual division within episodic autobiographical memory. We first review how these different episodic autobiographical representations (a conceptual and a perceptual representation) emerge from theories of autobiographical memory organization. We then describe neuroimaging findings that suggest that these forms of remembering map onto dissociable information processing systems in the brain. We also review work that shows how the anterior and posterior hippocampus facilitates activity within these large-scale processing systems. Finally, inspired by research on how episodic autobiographical memory serves a variety of non-memorial functions, including directing decisions and future behaviors (e.g., Pillemer, 2003; Alea and Bluck, 2007), we discuss decision-making scenarios that benefit from taking a perceptual vs. conceptual form of remembering. Here, we also note how distinctions in remembering may extend to other non-directive (self and social) autobiographical functions.

## Theories of Autobiographical Memory Access and Organization

When retrieving autobiographical experiences, episodic memory supports the ability to richly recall an experience as it occurred during a specific time and place (Tulving, 2002; Szpunar and McDermott, 2009). During retrieval, these episodic memory processes construct a detailed memory representation by associating different types of event information processed by disparate component systems (e.g., visual details, auditory details, conceptualized information; Greenberg and Rubin, 2003; Rubin, 2005; Moscovitch et al., 2016). This constructive feature of episodic memory means that multiple types of autobiographical memory representations can be formed by engaging different combinations—and relative weightings—of the component processes (Rubin, 2006; St. Jacques et al., 2011; Cabeza and Moscovitch, 2013; Moscovitch et al., 2016).

A theory of autobiographical knowledge organization suggests that one distinction in how autobiographical memory representations are formed is as primarily conceptual or perceptual episodic events. According to this theory, autobiographical event information is stored in a hierarchy, at different levels of abstraction (lifetime periods, general events, specific event, event-specific knowledge; Conway and Pleydell-Pearce, 2000; Conway, 2005). One possibility is that episodic information (i.e., details) about an event is simultaneously stored at different levels within this organization structure, with the conceptualized details (e.g., “I remember this event fondly as I was falling in love right then and there”) and contextualized perceptual details of the same memory stored separately (e.g., “We sat arm-in-arm on a picnic bench in Paris”; Conway and Pleydell-Pearce, 2000; Conway et al., 2016). This possibility leads to the theory that, depending on the reason for remembering the past (i.e., whether the conceptual vs. perceptual elements are emphasized at retrieval), disparate component processes will be engaged to activate the associated details (Burt et al., 2003).

**Figure 1:**
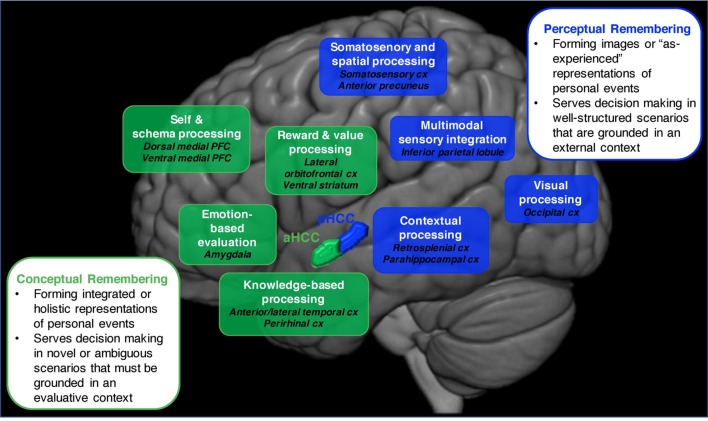
An illustration of the neural networks that support conceptual vs. perceptual forms of autobiographical remembering. The conceptual remembering network (depicted in green) is proposed to involve brain regions that are implicated in schematic (dorsal and ventral medial PFC), emotion-based (amygdala), reward and value-based (ventral striatum), and knowledge-based (anterior and lateral temporal cx, perirhinal cx) processing. This network is engaged *via* activation in the anterior hippocampus. The perceptual remembering network (depicted in blue) is proposed to involve brain regions that are implicated in contextual (retrosplenial cx, parahippocampal cx), somatosensory and spatial (somatosensory cx, anterior precuneus), visual (occipital cx) processing as well as regions implicated in multimodal sensory integration (inferior parietal lobule). This network is engaged *via* activation in the posterior hippocampus. Abbreviations: PFC, prefrontal cortex; cx, cortex.

In other words, a person can take different remembering strategies for autobiographical memory retrieval, an idea supported by classic research (e.g., Schank and Abelson, 1977; Reiser et al., 1985) as well as more recent findings (Brown, 2005; Ford et al., 2011; D’Argembeau et al., 2013; Sheldon and Chu, 2017). More specifically, we propose that there are different collections of component processes that will be activated and emphasized to different degrees to enable a conceptual or perceptual form of remembering. In the next section, we propose how this cognitive division is also reflected in patterns of neural activation (Figure 1).

## Distinct Neural Systems for Forms of Episodic Autobiographical Remembering

Autobiographical memory research has noted neural distinctions between remembering episodic (*That time I was funny*) or semantic (*I am funny*) autobiographical knowledge (Tulving, 1972; for more recent examples, see Burianova et al., 2010; Brown et al., 2018), yet fewer studies have looked at distinctions in different forms of *episodic* autobiographical memory. Our proposed division between conceptual and perceptual remembering assumes that different episodic memory details of a recalled event are used to form the underlying representation, which is reflected in the brain. Evidence for how this division is reflected in the brain come from a reported division within the default network—a collection of brain regions that overlaps considerably with the autobiographical memory network—that resembles conceptual and perceptual remembering (Buckner et al., 2008; Spreng et al., 2009; Andrews-Hanna et al., 2014). This research has described two cortical subsystems of the default network that access and process different types of self-generated information. One circuit, labeled the dorsal-medial subsystem, is involved in processing stored conceptual and schematic information related to a person’s experiences. The dorsal-medial subsystem is comprised of brain regions implicated in evaluative, schematic and gist-based processing [e.g., anterior and lateral temporal cortex, lateral orbitofrontal and anterior prefrontal cortex (PFC)], and is used to form abstracted representations of perceptual experiences (e.g., Yarkoni et al., 2008; Binder et al., 2009; Binder and Desai, 2011; Lin et al., 2016). Another circuit, labeled the medial-temporal subsystem, is involved in processing perceptual and imagery-based self-generated information. The brain regions involved in this circuit are those implicated in perceptual and context-based processing (e.g., retrosplenial cortex, parahippocampal cortex, inferior parietal lobule), allowing this subsystem to form mental event representations by reactivating what was externally experienced (seen, heard) during the event.

Other neural evidence for a neural division between conceptual and perceptual remembering comes from a model of memory that proposes similar subsystems to the default network for forms of recognition memory retrieval. This model proposes two mnemonic subsystems that emerge from the medial temporal lobes (MTLs) for accessing different episodic content (Ranganath and Ritchey, 2012; Ritchey et al., 2015; Reagh and Ranganath, 2018). There is an anterior temporal lobe subsystem that connects the region of the MTL implicated in conceptual processing (i.e., perirhinal cortex) to some of the regions found within the dorsal-medial subsystem for retrieving conceptual knowledge and those important for evaluating information [e.g., amygdala, ventromedial (vm) PFC]. There is also a posterior medial network that connects the region of the MTL implicated in processing external contextual information (i.e., parahippocampal cortex) to regions found within the medial-temporal subsystem that support retrieving specific situational elements of an encountered event (e.g., retrosplenial cortex) as well as perceptual processing (e.g., visual cortex).

We suggest that a similar subsystem division exists for retrieving the conceptual or perceptual episodic details of autobiographical memories and research has already begun to provide supporting evidence (Figure 1). There are studies comparing neural activity during different stages of autobiographical memory retrieval: during an early access vs. later elaboration stage of autobiographical memory. During the early access stage, higher-order information about an event is retrieved and evaluated, requiring the “conceptual system” of autobiographical remembering. During the later elaboration stage, the perceptual and experiential details of an event are accessed, requiring the “perceptual system” (Addis et al., 2007; St. Jacques et al., 2011; McCormick et al., 2015). Other autobiographical memory research has looked at neural regions that support retrieving general vs. specific autobiographical events, reminiscent of our proposed division between conceptual and perceptual remembering (Addis et al., 2004; Levine et al., 2004). One such study reported that retrieving autobiographical memories as specific events vs. personal knowledge commonly activated a number of regions, including the MTL, but that specific events recruited regions implicated in the perceptual subsystem (precuneus and superior parietal lobe) as well as self-reference regions (anteromedial PFC; Ford et al., 2011). Finally, in one of our recent experiments, we directly tested how the conceptual and perceptual subsystems would support remembering the same autobiographical memory in different ways. We ran a functional magnetic resonance imaging (fMRI) study in which participants focused on either the conceptual (the thematic or action details) or perceptual (the visual and contextual event details) elements of an autobiographical memory. Our key finding was that different neural networks, that aligned with what is presented in Figure 1, uniquely supported recollecting an event as a concept or percept (Gurguryan and Sheldon, submitted; for a related finding, see Martial et al., 2018). In the next section, we propose that these large-scale networks are systematically engaged by the anterior and posterior hippocampus to cue these details, determining if a memory is recalled conceptually or perceptually.

## Hippocampal Contributions to Forms of Episodic Autobiographical Remembering

During autobiographical memory retrieval, the hippocampus associates and integrates information from larger processing systems to access memory details to form a coherent mental representation (Nadel and Moscovitch, 1997; Hassabis and Maguire, 2009; Winocur and Moscovitch, 2011; Maguire and Mullally, 2013; Moscovitch et al., 2016; Sheldon and Levine, 2016; Sekeres et al., 2018). Traditionally, this role of the hippocampus in retrieval has been studied by considering the hippocampus as a homogenous structure, yet, there is mounting evidence that the anterior and posterior hippocampus perform distinct memory retrieval functions (Poppenk et al., 2013; Strange et al., 2014). For autobiographical memory, these functional distinctions along the hippocampal longitudinal axis are often interpreted with the gradient theory, such that the anterior and posterior hippocampus support accessing the coarse-grained vs. fine-grained details of a memory, respectively (Evensmoen et al., 2013; Collin et al., 2015; McCormick et al., 2015; Sheldon and Levine, 2015). The anterior hippocampal activity is thought to track accessing conceptual details of past personal memories (e.g., remembering that a conference took place at a waterfront Hotel) whereas posterior hippocampal activity tracks accessing and elaborating on fine-grained event details (e.g., recalling sitting next to Phife at the conference). There is other evidence that the anterior and posterior hippocampus are differently tuned towards representing novel vs. familiar (perceptual or experiential) mnemonic information. The anterior hippocampus is the center of a larger-scale novelty network for memory and responds to new interpretations of old events (Poppenk et al., 2010; Kafkas and Montaldi, 2018) whereas the posterior hippocampus is situated to respond to familiar perceptual and experiential information of an event (see Kondo et al., 2008; McCormick et al., 2015; Zeidman et al., 2015).

These discrepancies in hippocampal function allow for an event to be recalled for different reasons. Moreover, the reason for remembering an experience (expanded upon in the next section), signaled by prefrontal brain regions, is what determines the placement of activity along the hippocampal longitudinal axis to direct autobiographical memory retrieval (Preston and Eichenbaum, 2013; Rajasethupathy et al., 2015). If the purpose is to retrieve an episodic autobiographical memory conceptually, the anterior hippocampus will be preferentially activated to recruit the associated details *via* connected regions that process higher-order or coarse-grained information (e.g., the temporal cortices; gradient theory) as well as regions important for evaluative processing (vmPFC, ventral striatum). As such, the resulting memory representation will deviate from the initial encoding experience (a novel representation). If the purpose is to retrieve an episodic autobiographical memory perceptually, the posterior hippocampus will be activated so that the details from a memory are reinstated as they were initially experienced. Finer-grained perceptual details (gradient theory) that represent a close approximation of the encoded experience (a familiar representation) will be accessed *via* direct connections to regions that process and integrate somatosensory and perceptual information (e.g., parahippocampal, retrosplenial cortices, visual and somatosensory cortex).

Our model assumes that the anterior and posterior hippocampus—and the larger neural networks—are interconnected so there is an obligatory interaction between these processing systems when constructing an episodic memory representation (Sheldon and Levine, 2016; for related ideas, see Burke et al., 2018). In addition to suggesting that an autobiographical memory is not remembered as either/or a conceptual or perceptual episodic event, this idea also raises questions about the directionality of the functional connections between the hippocampal segments when remembering. There is a growing body of work suggesting that the anterior hippocampus plays a directive role in memory retrieval compared to the posterior hippocampus, particularly when forming complex mental event representation. For example, there is evidence that the anterior hippocampus is necessary for tasks that involve the online flexible construction of mental representations, including autobiographical experiences (McCormick et al., 2015; Ito and Lee, 2016; Mack et al., 2018; Monge et al., 2018), but not more rigid semantic memories that do not require this flexibility (e.g., Manns et al., 2003a,b; Winocur et al., 2010). With respect to our framework, it could be that a higher-order conceptual construct is a necessary framework for recalling autobiographical representations since recalling these events as episodic memories always require manipulating existing event-based knowledge (Nadel and Moscovitch, 1997; Moscovitch et al., 2006, 2016; Sekeres et al., 2018). This idea, however, is highly speculative and we bring it forward to stimulate research on understanding the ubiquitous role of the anterior hippocampus in the forms and functions of memory.

## The Functions of Conceptual and Perceptual Autobiographical Remembering

To this point, we have discussed how our model proposes that episodic representations of autobiographical experiences can be formed with predominately conceptual or perceptual information. Another chief element of our model is that the ability to form these different representations is to serve disparate functions outside the domain of remembering (Alea and Bluck, 2007; Vandermorris et al., 2013; Madore et al., 2016; Schacter et al., 2017; Mar and Spreng, 2018). One well-studied function for autobiographical memory is to direct future behavior, which includes cognitive tasks such as problem-solving, future thinking, and decision-making (Pillemer, 2003; Bar, 2009; Schacter, 2012).

An example of such a directive function is making memory-based decisions: problems that require accessing information from an associated past memory. These decision problems can present as open-ended or close-ended tasks (Simon et al., 1987). Open-ended tasks are those with uncertain decision outcomes and/or multiple ways for an outcome to be reached, such as deciding on home renovations or how to plan a party. Close-ended tasks are those that have a set path that indicates a certain outcome, such as a plumber relying on a structured set of actions to decide how to fix a broken toilet. One key difference between these decision scenarios is that open-ended tasks are less reliant on the situation (i.e., environment) they occur in than close-ended tasks. Open-ended tasks will vary across situations (how you renovate a home will change as a function of the home) whereas close-ended decisions are more likely to occur similarly across situations (i.e., the way you fix a toilet is similar across bathrooms). This distinction is important for understanding when perceptual vs. conceptual remembering will be most effective in guiding decision-making.

Since closed-ended decisions are more structured and tied to the environment, perceptual remembering will be effective to use information from a person’s current surrounding as a cue to access a relevant past memory. This is helpful to make rapid decision about encountered stimulus (e.g., “Is this animal or food safe or dangerous?”), navigation tasks (recalling the precise path to get from point A to B) or recalling the location of a lost item (e.g., “Where did I put my keys?”). Using perceptual remembering for these decisions will recruit the posterior hippocampus to activate perceptual and experience-based processes to mentally reinstate a past experience and apply it to the current situation. Perceptual remembering, however, will be less useful for open-ended decisions. This is because external (perceptual) cues from a person’s current surrounding may not re-activate the correct past memory to gather information needed to make a decision. In these cases, conceptual remembering is better suited because this form allows an individual to access generalized memory representations and evaluate them as they apply to the new, open-ended decision situation. Examples of such decisions are novel and “noisy” problems that first require generating a desired outcome (e.g., “How should I redecorate this bathroom?”), and then using this internally represented goal/outcome (concept) to retrieve relevant past memories (e.g., other home improvement project undertaken in the past). In some of our previous work, we found that open-ended social problems (e.g., making new friends) require episodic simulation to construct solutions to these problems (Sheldon et al., 2011; Vandermorris et al., 2013), which we posit is based on conceptual remembering creating new outcomes to these problems. Using conceptual remembering will call upon the anterior hippocampus during remembering which will stimulate activity in brain regions implicated in schematic and evaluative processes (e.g., vmPFC; Euston et al., 2012).

**Figure 2:**
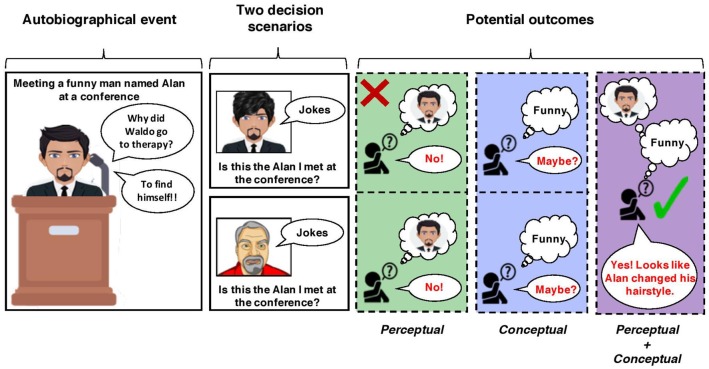
A schematic depicting an example of decision-making scenarios in which using only conceptual or perceptual forms of remembering lead to a correct (but uncertain) or faulty outcome. In this example, a “decision-maker” met a funny individual with short hair and a beard, named Alan, at a conference (left panel). In one scenario, the decision-maker later encounters someone who looks like Alan but with a different hairstyle (top row). The decision-maker must use their memory of Alan from the autobiographical event to decide if this encountered individual is him. If they recall the autobiographical event *via* perceptual remembering, by rigidly retrieving every detail about how Alan looked, they will not correctly identify this individual as Alan because his hairstyle (i.e., a perceptual detail) has changed. If they recall the autobiographical event *via* conceptual remembering, they will more likely be able to identify him as Alan, even with his new hairstyle; however, this decision will lack certainty (i.e., this is *maybe* Alan). In another scenario, the decision-maker later encounters a man with a similar beard and hairstyle to Alan (bottom row). Like above, if they recall meeting Alan *via* perceptual remembering, they will correctly decide that this new man is not Alan. If they recall this event conceptually (i.e., only recalling they met a funny man the conference), they may accidentally identify this new man as Alan because of one overlapping feature (being funny) represented at the concept level.

Although there may be certain situations that benefit from representing our past as primarily concepts or percepts for directive functions of autobiographical memory, the ability to dynamically shift between these forms of remembering is likely what underlies the optimal use of memory (also see, Richards and Frankland, 2017; Duncan and Schlichting, 2018). This idea becomes clear when thinking about the potential errors during decision-making that would arise if only one method of remembering were used, which is illustrated in Figure 2. Following this figure, relying primarily on conceptual remembering can lead to applying autobiographical memories too broadly (i.e., liberally) because other pertinent details from an experience are ignored (e.g., meeting a short-haired and bearded individual at a conference who told funny jokes). This can lead to information from past experiences to be incorrectly applied to a current decision-making scenario and a poor outcome (e.g., mistakenly identifying other funny, short-haired, and bearded individuals as that person from the conference). On the other hand, an over-reliance on perceptual remembering can cause autobiographical knowledge to be applied too conservatively (i.e., rigidly), leading also to decision-making errors. If a person is searching for information from a past experience to decide solely by focusing only on specific perceptual details may obscure the ability to correctly locate a memory to inform their decision (e.g., deciding that the funny individual you are speaking to, whom you did meet at a previous conference, is *not* that individual because they changed their hair-style). In other words, rigidly adhering to only one form of remembering can lead to information from the past to be incorrectly applied to a current decision-making scenario. Instead, shifting the relative contributions of how we remember is ultimately what allows autobiographical memories to serve several adaptive functions. Given the central role of the hippocampus in these forms of episodic remembering, it is likely that this brain region is the key to this adaptivity.

In this final section, we emphasized how our framework describing different forms of remembering impact memory-based decision-making, however we predict that this impact would present similarly for other directive functions of autobiographical memory, including planning future behaviors and solving personal problems. Outside the directive functions of autobiographical memory are those that relate to the self and to social functions. Existing research has provided views on how accessing autobiographical memories at different levels, similar to conceptual and perceptual remembering, can benefit and impair these functions (e.g., Pillemer, 2003; Alea and Bluck, 2007; Prebble et al., 2013). Although beyond the scope this article, it is worthwhile to pursue research aimed at understanding how different forms of remembering operate across these functions.

## Conclusions

Autobiographical memories are complex constructs that encompass a rich array of information, including conceptual and perceptual episodic details. A single past experience can be represented at retrieval in a variety of ways, depending on how these details are accessed, and this is determined by the goal of remembering. Here, we provided a brief overview of theoretical accounts and empirical findings on autobiographical memory organization and retrieval to suggest a new division in the episodic autobiographical remembering. We proposed two forms for remembering the past—as a concept or as a percept—and provided a neural account for these different forms of remembering, emerging from disparate hippocampal-cortical networks. We defined the reason for these forms of remembering by describing their functional roles in decision making, providing a new outlook on the way the goals of a current task benefit from the flexibility of the episodic autobiographical memory. Finally, we proposed that the ability to shift between different forms of remembering, specified by the relative contribution of the outlined hippocampal-cortical networks, is the key to adaptive memory.

## Author Contributions

SS constructed the presented perspective and outline for the article. SS, LG, and CF contributed to writing the final draft and provided editorial feedback.

## Conflict of Interest Statement

The authors declare that the research was conducted in the absence of any commercial or financial relationships that could be construed as a potential conflict of interest.
